# From Black Box to Machine Learning: A Journey through Membrane Process Modelling

**DOI:** 10.3390/membranes11080574

**Published:** 2021-07-29

**Authors:** Claudia F. Galinha, João G. Crespo

**Affiliations:** LAQV-REQUIMTE, Department of Chemistry, NOVA School of Science and Technology, Universidade NOVA de Lisboa, 2829-516 Caparica, Portugal; jgc@fct.unl.pt

**Keywords:** membrane processes, modelling, multivariate data analysis, artificial intelligence, chemometrics, fluorescence excitation-emission matrices (EEM), big data, PLS, ANN, PCA

## Abstract

Membrane processes are complex systems, often comprising several physicochemical phenomena, as well as biological reactions, depending on the systems studied. Therefore, process modelling is a requirement to simulate (and predict) process and membrane performance, to infer about optimal process conditions, to assess fouling development, and ultimately, for process monitoring and control. Despite the actual dissemination of terms such as Machine Learning, the use of such computational tools to model membrane processes was regarded by many in the past as not useful from a scientific point-of-view, not contributing to the understanding of the phenomena involved. Despite the controversy, in the last 25 years, data driven, non-mechanistic modelling is being applied to describe different membrane processes and in the development of new modelling and monitoring approaches. Thus, this work aims at providing a personal perspective of the use of non-mechanistic modelling in membrane processes, reviewing the evolution supported in our own experience, gained as research group working in the field of membrane processes. Additionally, some guidelines are provided for the application of advanced mathematical tools to model membrane processes.

## 1. Introduction

Typically, membrane processes require close monitoring to assess the performance of separation and ensure the quality and characteristics of the fractions achieved. Additionally, and independently of the system studied, the monitoring of membrane performance and of fouling formation is usually an essential requirement to make decisions about membrane cleaning procedures and thus, to maximize the lifespan of membranes. In this context, modelling of the membrane process is indispensable, since it allows to simulate (and predict) membrane performance, to infer about optimal process conditions, to correlate experimental measurements with fouling potential and fouling development, to control the process (in particular when online monitoring data is available), to increase the knowledge about the process and ultimately for process design.

In general, when applied to membrane processes, mathematical models can be developed to describe the filtration process (e.g., permeability, selectivity) through well-known physical equations that describe the mechanisms involved in the membrane process. Some models aim to describe variables that cannot be assessed experimentally (or are difficult to assess) and correlate them with mensurable variables (usually performance variables), disclosing the mechanical behavior of the process. When these models are regulated by physicochemical laws, they are usually called mechanistic or deterministic models. Indeed, there are many possible ways to classify mathematical models [1], but in this work, only the mechanistic and non-mechanistic classification will be discussed.

However, besides the mechanistic models, based on known physicochemical equations, non-mechanistic models can also be used in membrane processes to describe the performance and behavior of the systems. Such models are based on experimental data and sometimes are called empirical models; however, when models are developed based on statistical tools, they are also called statistical models. For the purpose of the present work, non-mechanistic models are defined as data-driven models based on mathematical tools, implemented through computational algorithms. These non-mechanistic models are also commonly called black-box models due to the character of the mathematical correlation (sometimes unknown or hidden in complex algorithms), which is not driven from physicochemical insights. However, nowadays, terms as Chemometrics, Machine Learning, and Artificial Intelligence are also commonly used to describe the use of such mathematical/computational tools and are usually recognized as valuable in different areas of science.

Multiparametric non-mechanistic modelling tools usually involve principal components analysis (PCA), or one of its several variations, multilinear regressions, such as projection to latent structures (PLS) regression (also called partial least squares regression) or artificial neural networks (ANN), among other tools. Independently of the modelling tools used, machine learning techniques require computational algorithms, a significant amount of data, have statistical parameters to assess validity, and correlate a group of parameters (called inputs) to disclose the key variables selected to be modelled (the outputs). Such methods also require a calibration (i.e., the learning of the mathematical structure), where the algorithms are trained using experimental data (both input and output parameters assessed experimentally are required) in order to define the models. After the calibration, a step of validation (also called test) with a new set of experimental data (not used for calibration) is required. The models achieved can then be used to predict the outputs based only on the input parameters.

Despite the actual dissemination of terms as Machine Learning and Artificial Intelligence, just a few years ago, the use of such computational tools to disclose correlations between different variables when modelling membrane processes was not very common and usually regarded as not useful from a scientific point-of-view, lacking physicochemical meaning, and not contributing to the understanding of the phenomena involved. This work aims at providing a personal and historical perspective of the use of non-mechanistic modelling in membrane processes. Furthermore, the present work aims at showing how advanced mathematical tools (non-mechanistic models) can be used for the modelling of membrane processes to solve practical problems (such as process monitoring and fouling development) and the advantages of using such tools in situations where mechanistic models are not sufficient because the understanding of the bio/physical phenomena is incomplete.

## 2. Non-Mechanistic Modelling

The work developed using conventional mechanistic modelling of membrane processes is huge and comprises different membrane types and membrane processes [2]. In fact, these models and modelling systems are useful and non-replaceable; however, there are situations where the knowledge about physicochemical phenomena and interactions is not sufficient to allow the development of successful and useful models. Furthermore, when dealing with large and complex amounts of data resulting from monitoring a membrane process, the use of computational tools is essential to extract meaningful information, able to be used in an insightful way. While mechanistic modelling aims at describing the system (and the experimental data) based on a priori knowledge, non-mechanistic modelling aims at finding the system phenomena and dynamics that are inherently contained in the experimental data.

Multivariate data analysis aims at study data sets composed by several variables that describe the system, and different multivariate methods can be used with distinct objectives. For data mining and non-mechanistic modelling, several mathematical tools are available and can be used, alone or in combination, depending on the data available and the objectives of modelling. Some multivariate data analysis methods are called unsupervised and aim at finding variance within data to identify patterns and/or groups (or clusters) in data sets. For example, principal component analysis (PCA) is a non-supervised modelling tool, able to transform the initial variables into independently linear combinations, called principal components (PC). PCA decomposes a matrix of data (*X*) into a product of two new matrices with reduced dimensionality, the scores matrix (*C*), having the same number of rows and as many columns as PCs, and the loadings matrix (*P^T^*), with the same number of columns of *X*, plus a “noise” matrix (ε), containing the data variance not explained by the number of PC used in decomposition.

*X* = *C* · *P^T^* + ε (1)

In multivariate regression methods, two or more input variables are correlated to predict an output variable, in the same way of a regression between two variables. Multivariate regression tools may include linear or non-linear functions to correlate the input variables with the output and are called supervised modelling tools, due to their ability to learn simultaneously from a *X* matrix (inputs) and an *Y* matrix (outputs), correlating both sets of data, and aiming at predicting the outputs (*Y*). Projection to latent structures (PLS) modelling is a multivariate regression technique using linear correlations (multilinear) related with PCA, where the variables space is reduced and the covariance between the matrices *X* and *Y* maximized. Therefore, redundancy in input and output data is eliminated, allowing higher robustness when there is collinearity and noise in the experimental data sets.

While PLS modelling results in multilinear regressions between the inputs, to predict the outputs other nonlinear mathematical tools are available. Artificial neural networks (ANN) are well known algorithms organized in nodes (inputs, hidden layer(s) and outputs), as a representation of brain neurons, where each node contains a mathematical function. ANN modelling is based on patterns, within the neural network structure, and can be used to reveal complex nonlinear correlations between inputs and outputs.

An example where the non-mechanistic modelling is useful to extract some meaningful information from a complex data set is when using spectroscopic techniques for process monitoring, which may result in large sets of data (the spectra). In these cases, the spectra acquired are not always easy of interpretation, especially when the system monitored is complex and prone to interferences, requiring data-mining techniques to extract rapidly meaningful information. The advantage of using computational methods to extract information from these data sets is obvious and usually well accepted by users, since they permit to recover information which otherwise would not be possible to assess.

**Figure 1:**
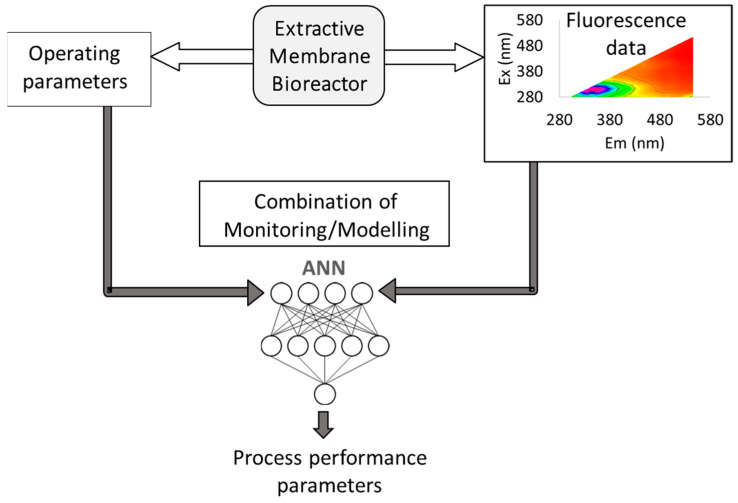
Schematic representation of using 2D fluorescence spectroscopy and operating parameters as inputs of artificial neural networks (ANN) to predict process performance parameters in an extractive membrane bioreactor (EMB).

Aiming at monitoring an extractive membrane bioreactor (EMB) for the degradation of chlorinated organic compounds, operating with mixed cultures, Wolf et al. explored in the early 2000 decade the use of two-dimensional (2D) fluorescence spectroscopy, which provides 3D spectra (excitation-emission matrices, EEMs) by scanning simultaneously a range of different excitation and emission wavelengths, in combination with artificial neural networks (ANN) [3,4,5]. In membrane processes involving biological reactions, there are several parameters to monitor, to assess both membrane and biological performance, as well as phenomena occurring due to the interaction of the biological media with the membranes (e.g., biofilm and/or fouling formation). Therefore, Wolf et al. used artificial neural networks (ANN) to extract the information relative to biofilm developed at the membrane surface comprised in the complex fluorescence matrices obtained on-line, in real time, during the EMB operation (Figure 1).

Machine learning, and in particular ANNs, were a novelty as modeling tools in biotechnology and medical applications in the beginning of the XXI century [6], particularly interesting due to the complex character of biological interactions, the high number of compounds involved and non-linearity of biological systems. Furthermore, spectroscopic techniques as analytical and fingerprinting tools were also being widely explored to characterize biological media, as reviewed by Pons et al. [7]. In particular, 2D fluorescence spectroscopy was studied by Scheper and co-workers as a monitoring tool for microbial processes [8,9,10]. Thus, it is not surprising that the use of these same tools was extended firstly to monitor membrane processes involving biological reactions, as done by Wolf and co-workers.

## 3. Concerns about “Black Box” Modelling and Precautions Required When Using This Approach

While the use of non-mechanistic modelling to deconvolute spectral data offers a clear advantage, the use of the same modelling tools to correlate operating variables with performance parameters raised several concerns among the scientific community.

The most evident concerns are related with the validity of the mathematical correlations, since they are not based in physicochemical phenomena and require calibration with experimental data, being very susceptible to variations of the conditions used during calibration (the extrapolation ability of such models is limited). Problems related with data selection and preparation prior to the calibration process (or “learning”), the selection of internal structures (latent variables in PLS or number of nodes in ANN) of mathematical algorithms, and proper validation of the correlations achieved can result in overfitted models, with poor predictive ability, or misleading correlations, where random values are wrongly correlated. However, pitfalls in calibration and validation common to non-expert users can easily be avoided and evaluated by following good modelling practices.

**Figure 2:**
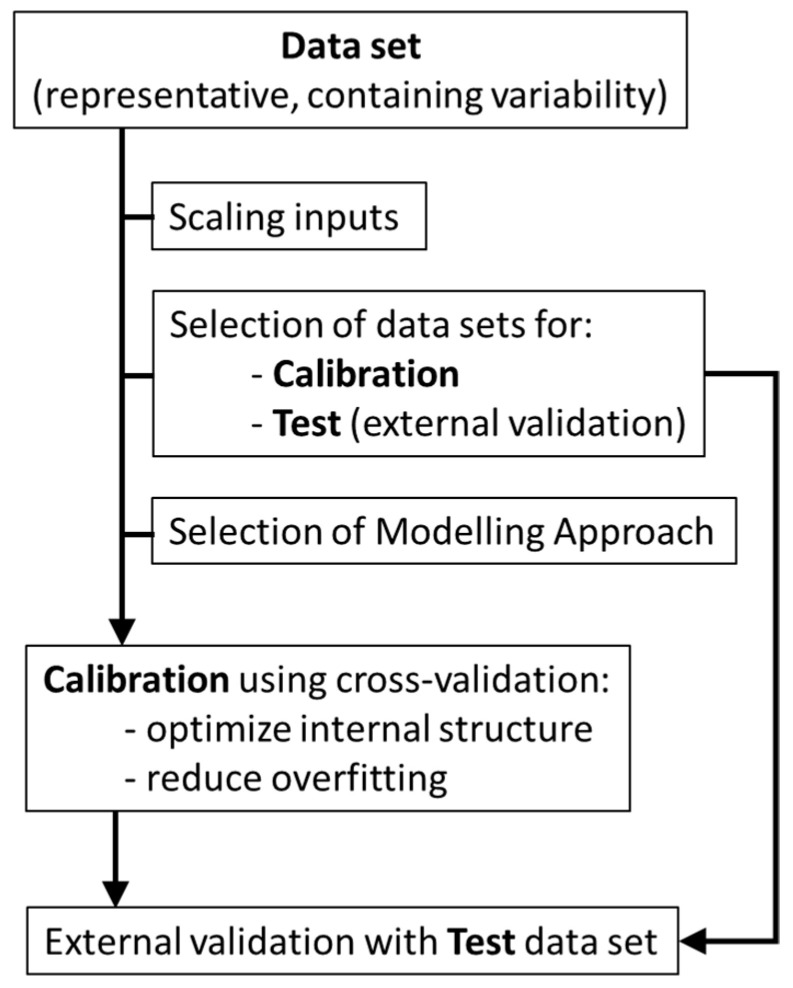
Diagram summarizing non-mechanistic modelling procedure.

As shown in Figure 2, the first step to apply non-mechanistic models consists in selecting a good data set to be used for non-mechanistic modelling. The data set needs to be representative of the system studied, using significant parameters (variables) to describe the system at hands, both for inputs and outputs. Furthermore, each variable should contain enough variability, to allow the mathematical structure to “learn” about the system behavior (i.e., how variations in selected parameters correlate with the process status). An additional step is often required to assure that the inputs used have the same initial weight, avoiding that input parameters with higher values (independently of variance captured) have more impact in the modelling structure than the other parameters. Although different scaling procedures can be used, this step is usually performed to normalize the range of each variable (*x*), according with the following equation:

(2) $$x^{\prime }=\frac{x-\bar{x}}{\sigma_{x}}$$

The standardized values (*x*′) are calculated based on average and standard deviation found for each parameter ($\bar{x}$ and $\sigma_{x}$, respectively). Thus, the variance across each input variable is enhanced when related to changes in system performance.

Before calibration of a modelling structure, it is also needed to ensure that a good test data set is available for final validation of the model achieved. This data set can be selected randomly from the initial data set or acquired afterwards. Nevertheless, it should comprise data in the same range of conditions of the calibration set because non-mechanistic models are valid only within calibration conditions and comprise enough variability, to test the model in all range of calibration.

The selection of the most appropriated modelling tools is also an important step. For example, avoiding non-linear correlations when the system is linear or there is not sufficient data has a higher success in achieving good models by decreasing the possibility of data overfitting. Additionally, the assessment of the calibration procedure can be done through cross-validation. Cross-validation is usually performed by leave-one-out or leave-one-batch-out techniques, aiming at assessing how the use of different sub-sets of the calibration set performs to estimate the data left out. Cross-validation is performed sequentially across the entire calibration data set and allows to optimize the internal structure of the algorithm and minimize data overfitting [11].

Beyond the importance of the external validation using the test data set, it is imperative to assess accurately the quality of fitting and estimation ability of the models achieved. Therefore, other critical aspect when using non-mechanistic modelling is the use of adequate parameters to infer about the quality of the models. Such evaluating parameters should include average errors for calibration, cross-validation, and test sets (usually through root mean squared error: RMSEC, RMSECV, and RMSEP, respectively), as well as fitting ability, as calculated by the coefficient of determination (R^2^) for the same data sets. Additional statistic tests (e.g., Akaike information criterion [12]) are also useful to assess the quality of the models, although their use requires usually larger data sets and initial data following specific statistical behavior, such as Gaussian distribution.

Besides mathematical considerations about the unreliability of non-mechanistic methods, easily overcome with adequate modelling practices and evaluating parameters, a common criticism of such models is that as “black box” models, they do not lead to a better understanding of the physical phenomena involved in the process, having a limited application. However, the analysis of functions used in the computation algorithms can be in fact used to gain knowledge with the correlation found. This can be done by assessing the equations resulting from tools such as PLS regression (also called grey box, because they provide equations) or through sensitivity analysis of the input parameters related with the output(s).

In PLS regressions, the equation used to estimate an output (*y*) is based on the multilinear regression of input variables (*x*):

(3) $$y=b_{0}+b_{1}x_{1}+b_{2}x_{2}+\cdots +b_{v}x_{v}$$

Based on this type of equation, it is possible to determine for each output (*y*) the contribution (i.e., the weight (*z*)) of each input (*v*), using standard deviation ($\sigma_{v}$) and the regression coefficients (*b_v_*) of all variables:

(4) $$z_{v}=\frac{|b_{v}|\cdot \sigma_{v}}{\sum_{v=1}^{V}\left|b_{v}\right|\cdot \sigma_{v}}$$

For the ANN, the impact of each input can be assessed through a sensitivity analysis of each output value followed by an average for all observations. Sensitivity is calculated for each value by determining the normalized ratio between the output variables and input parameters [3].

The knowledge about which inputs are relevant in the prediction of outputs, as well as their relative weight in the model, provides a mechanistic insight about the processes being modelled. In fact, several studies aiming at modelling membrane processes (e.g., [3,13,14]) also provided this type of mechanistic insight, providing more than a useful tool for prediction, as it is discussed in Section 5.

## 4. Hybrid Modelling

**Figure 3:**
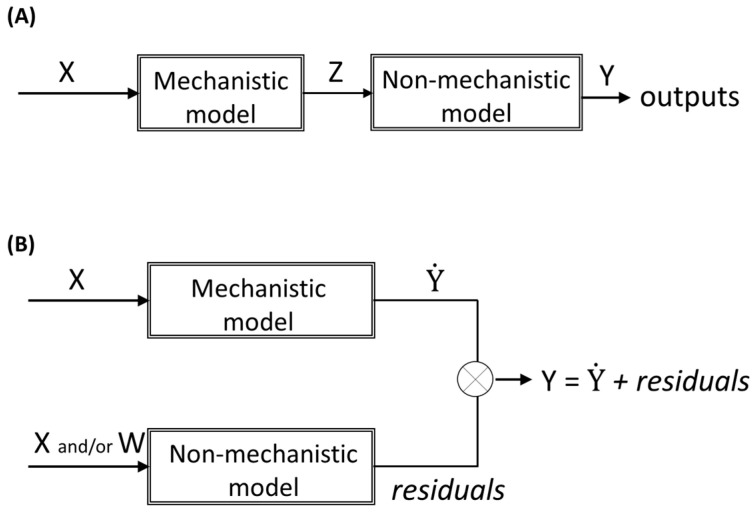
Hybrid modelling structures: (**A**) serial; (**B**) parallel.

Hybrid modelling refers to the combination of mechanistic and non-mechanistic models to model a specific system or process. The way each type of functions (mechanistic or non-mechanistic) interacts in hybrid models can be different regarding the modelling objectives. Usually, hybrid model structures can be classified as parallel or serial (Figure 3). While in serial configuration the non-mechanistic model is typically used to estimate the inputs required to the mechanistic model (Figure 3A), in parallel configuration, both mechanistic and non-mechanistic models are fed simultaneously by inputs (in parallel). Typically, in parallel configuration, the non-mechanistic model aims at predicting the deviation of the mechanistic model (the residuals), and thus, by combining both models, the mechanistic model is corrected to improve the output prediction. The rationale behind this hybrid modelling approach is that the residuals from the mechanistic model are not noise; they comprise information that can be uncovered with the non-mechanistic model. Furthermore, the description of the residuals from the mechanistic model can be achieved using the same inputs used in the mechanistic model (X) and/or using additional inputs (W), able to describe the missing information (Figure 3B).

**Figure 4:**
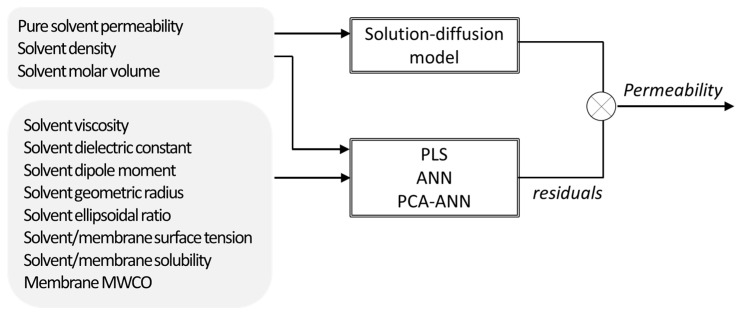
Hybrid modelling structure used for prediction of solvent permeability through nanofiltration membranes (PLS—projection to latent structures; ANN—artificial neural network; PCA—principal components analysis).

Santos et al. [15] assessed the use of mechanistic, non-mechanistic, and hybrid modelling strategies to describe the solvent transport through nanofiltration membranes. Since most used mechanistic models developed to the date were not sufficiently general to cover a wide range of membrane–solvent systems, alternative membrane and solvent descriptors were selected for that work, which were incorporated as inputs in the modelling structure alternatively through PLS regression, ANN, and PCA combined with ANN (Figure 4). In this parallel hybrid approach, the non-mechanistic models were used to estimate the deviations of the solution-diffusion model by incorporating the descriptor parameters as inputs. The hybrid modelling resulted not only in a better description of the experimental results (collected from several publications) but also allowed to identify which variables were more relevant for process performance, namely the ones whose contribution was not considered by the solution-diffusion model. Hence, the analysis of the input contributions in the PLS equation found that the mechanistic model used lacks further relevant information about solvent polarity.

In a different membrane process, hybrid modelling was also used to improve the modelling ability of an activated sludge model (ASM), commonly used to assess biological performance in wastewater treatment systems, applied to a membrane bioreactor, with minimal additional monitoring effort [16]. In the study, a parallel hybrid approach was used to improve the prediction of suspended solids, chemical oxygen demand in effluent and nitrate plus nitrite in effluent. Since the ASM used was calibrated with initial conditions (from first 50 days of operation) and performed for 400 days of operation without further recalibration, the ASM lacked detailed and real-time information from wastewater and operating parameters. The aim of the work was to improve the model prediction without using laborious analytical experiments, thus, 2D fluorescence spectroscopy data, collected directly in wastewater, biological media, and effluent, were used with a PCA plus PLS regression modelling approach to estimate the residuals from the ASM. The hybrid model allowed the improvement of ASM by incorporating fluorescence data, which can be obtained from monitoring the process in real time. Furthermore, adding operating conditions and analytical data together in the hybrid modelling did not significantly improve the predictions, showing that monitoring data from 2D fluorescence spectroscopy is sufficient to capture the variations in media composition lacking in the mechanistic model.

**Figure 5:**
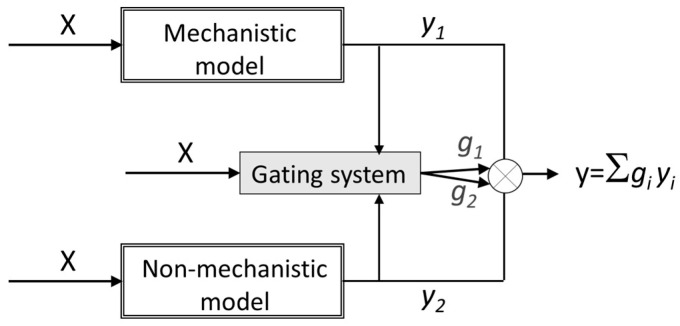
Parallel hybrid modelling using a competitive mixture-of-experts structure, where a gating system selects the contribution of each model to the final prediction.

In other study [17], hybrid modelling was used for prediction of counterion fluxes across an ion-exchange membrane in a membrane-supported biofilm reactor. The hybrid strategy was used to account for the complex biofilm contribution to the transport. In that work, besides the parallel hybrid strategy, where the inputs were also used with PLS regression to estimate the residual of the mechanistic transport model, a competitive mixture-of-experts structure was used (Figure 5). The mixture-of-experts structure used both mechanistic and non-mechanistic models to predict the counterion mass-transfer across the membrane and a gating system mediating the use of one or the other model based on the contribution of the inputs (the operating conditions) for the prediction of the output. By using the parallel approach, the PLS regression model was able to capture the information missing in the mechanistic model from the process operating data. However, for some counterions the prediction was unsatisfactory due to error variance included by the mechanistic model in the PLS calibration. Therefore, the limitations found with the parallel structure were avoided with the competitive mixture-of-experts structure being this modeling strategy chosen as a better choice in this application.

As shown for these membrane processes, hybrid models allowed a better understanding of the process than non-mechanistic models, not only because the known physical relations were used through a mechanistic model but also because by incorporating non-mechanistic models, it is possible to detect when or why the mechanistic model fails, identifying the type of information that is missing in mechanistic modelling (through the new inputs selected or different correlations).

Furthermore, such models are useful to reveal when the complexity of the system modelled is higher than the mechanistic models available. Therefore, hybrid modelling can be used in situations where the mechanistic models are not enough to describe the system, due to the complexity of interactions or by extending the use of models to operating conditions beyond the system assumptions (required for the mechanistic models). On the other hand, non-mechanistic modelling usually requires a relatively large amount of experimental data and has a limited extrapolation potential, which may also be overcome with the incorporation of a mechanistic model (even if using simplified assumptions), in a hybrid modelling structure.

However, it should be noted that when a mechanistic model is not enough to describe a system and hybrid modelling can be used, the assessment of using only non-mechanistic modelling should be performed. The use of non-mechanistic models can in fact simplify the mathematical correlations (by directly correlating the input parameters with the outputs) without requiring different functions (as in hybrid models) to monitor the process. Therefore, before following a hybrid modelling approach, the objectives of the modelling work should be well defined, and the use of only mechanistic and only non-mechanistic modelling should be first considered.

Besides their utility, the combination of non-mechanistic with mechanistic models increases the acceptability by users since it is easier to understand the mechanical concepts and the limitations causing the need for adding non-mechanistic models.

## 5. When Non-Mechanistic Modelling Is Seen as Learning

When aiming at modelling membrane processes, the requirement for using non-mechanistic modelling can firstly be originated from the need to incorporate non-conventional inputs as system descriptors, such as spectroscopic data, online analytical data, or system properties/operating conditions (as by using hybrid modelling, discussed in Section 4), or to deconvolute complex, large data sets resulting from system monitoring (as mentioned in Section 3). 

**Figure 6:**
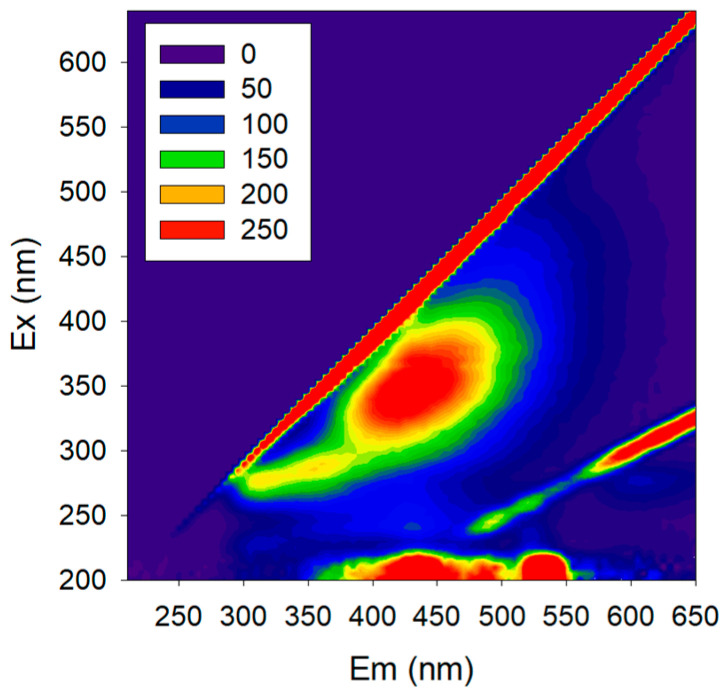
Fluorescence excitation-emission matrix (EEM) of a microbiological media plotted as a contour plot. The axes represent the wavelengths of excitation (Ex) and emission (Em) in nm, and the intensity of emission (in A.U.) is shown by color scale.

In fact, our journey in non-mechanistic modelling started with the objective of using 2D fluorescence spectroscopy to monitor membrane processes involving biological reactions. 2D fluorescence is a tool able to capture a large amount of information about biological systems (due to the several natural fluorophores present, e.g., amino acids, NADH, pigments) without disturbing the system (Figure 6). Fluorescence excitation–emission matrices (EEMs) can be acquired with an optical probe to assess the process directly from the media without sampling, and the probe can also be oriented towards the membrane surface, collecting data directly from it. The fluorescence spectra reflect not only the fluorophores present but also other media properties, since this technique is sensitive to the presence of several compounds which interact with the fluorophores, changing their fluorescence response, and/or with excitation and emission light (e.g., inner-filter effect). While a large amount of data is assessed by 2D fluorescence, the EEMs acquired are complex, and useful information cannot be extracted directly due to the several interferences present. Thus, non-mechanistic modelling should be used to extract meaningful information not only from fluorophores but also from the interferences, as they reflect the presence of other compounds.

When using non-mechanistic modelling to deconvolute fluorescence spectra applied to membrane processes monitoring, different modelling approaches were already studied. Firstly, and more intuitively, the mathematical tools were applied to correlate 2D fluorescence with compounds concentration or process performance parameters. 

When modelling an extractive membrane bioreactor (EMB) for the degradation of chlorinated organic compounds, 2D fluorescence spectra acquired in different positions of the membrane surface, where a biofilm develops, were used as inputs in ANN to estimate the concentration of 1,2-dichloroethane, ammonia and chloride in the bioreactor liquid medium [3]. The use of ANN was selected in this work after analyzing the possibility of spectra subtraction and concluding that a nonlinear technique based in pattern recognition would be better for fluorescence spectra deconvolution.

In membrane bioreactors (MBR) for wastewater treatment, a similar approach was also used correlating 2D fluorescence spectra acquired in the influent wastewater stream and in effluent permeate stream to predict chemical oxygen demand (COD) respectively in each stream [18]. In this study, projection to latent studies (PLS) regression was used as the non-mechanistic modelling tool. This tool was selected for being a multilinear tool, less complex than non-linear ANN, which can minimize overfitting and results in equations easier to interpret.

Nevertheless, besides estimation of concentrations in liquid media, both studies showed how such non-mechanistic models can help in elucidating system mechanisms [3,18]. The regression coefficients of the PLS multilinear equation can be used to assess the weight of each input parameter (i.e., each excitation/emission wavelength pair) and hence the contribution of each part of the fluorescence spectra, for the prediction of each output compound. While when using ANN, a sensitivity analysis can be used to highlight which input parameters most significantly influence the prediction of the output parameters.

The non-mechanistic deconvolution of fluorescence spectroscopy in both type of biological membrane reactors, extractive membrane bioreactor (EMB) and membrane bioreactor (MBR), were also subject to improvement through the use of principal components analysis (PCA) to compress the fluorescence data set into a smaller number of variables (principal components, PC), prior to correlating it with process performance [5,19]. Such approach simplifies and reduces the number of inputs to be used for models learning, regardless of the modelling tool used (ANN or PLS), reducing the computational effort. Furthermore, it allows the simplification of the correlations and can deal a priory with noise removal, since only the main variance captured in the fluorescence spectra are used in the final correlations.

**Figure 7:**
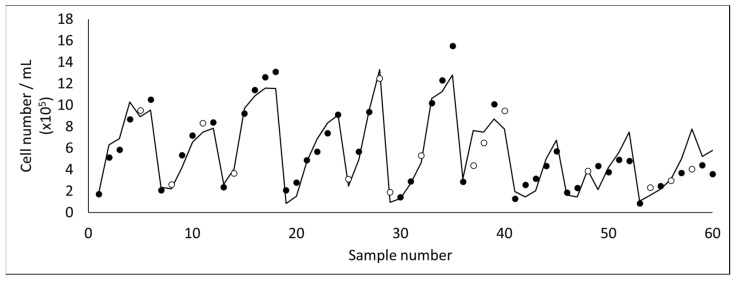
Experimental data (black circles for training and open circles for test) and model prediction (line) of microalgal cell concentration during ultrafiltration experiments and cell growth.

**Figure 8:**
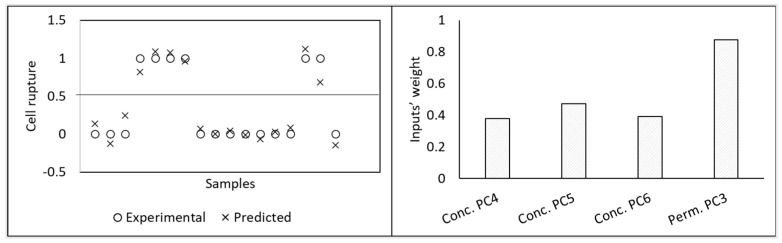
Results of PLS modelling for prediction of microalgal cell disruption during harvesting. Left: Experimental (circles) and predicted (x) values for cell rupture (unbroken (<0.5) and disrupted (>0.5) cells). Right: Weight of inputs selected (respectively, principal component (PC) 4, 5, and 6 from fluorescence spectra acquired in concentrate and PC 3 from fluorescence spectra acquired in permeate).

Using the methodology of PCA followed by PLS, 2D fluorescence was posteriorly used to develop a monitoring tool for the harvesting of microalgae (*Dunaliella salina*) by membrane micro- and ultrafiltration [20,21]. These studies focused mainly on monitoring cell concentration (Figure 7) and cell integrity, due to the fragility of the microalga assessed. Cell rupture occurs easily in *D. salina*, resulting in the release of valuable intracellular compounds to the water media (which are then lost in the permeate). Due to the current difficulty on analyzing the concentration of intact cells and assess the integrity of the microalga in real time (usually optical microscopy or flow cytometry techniques are required), the aim of these studies was to develop a monitoring tool able to be used online, with an optical probe to assess fluorescence, in order to decide at real time when to stop the filtration process, assuring cell integrity. Additional computational tools were used to select the most useful inputs from the original data set. Therefore, as can be seen in Figure 8, from the 20 principal components (PC) resulting from PCA to spectral data (10 PCs from fluorescence acquired in the concentrate and 10 PCs from fluorescence acquired in the permeate) only 4 were selected as useful for predicting microalgae cell disruption, with different weights [20].

**Figure 9:**
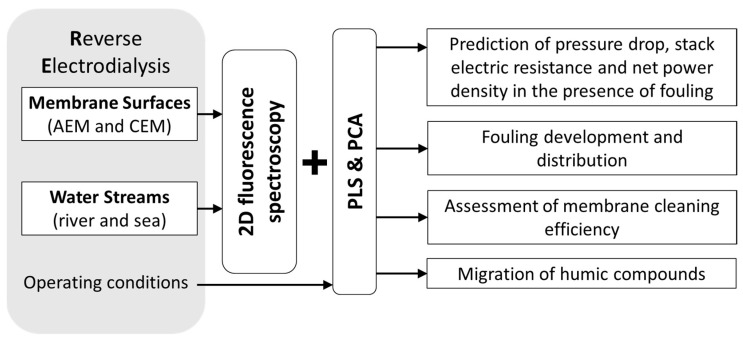
Applicability of 2D fluorescence spectroscopy combined with non-mechanistic modelling (projection to latent structures and principal component analysis) for the monitoring of a reverse electrodialysis (RED) process (AEM—anion exchange membrane; CEM—cation exchange membrane) [22,23].

Besides the characterization of membrane processes involving biological reactions, PCA was also used to extract information (qualitative information) from fluorescence scans of membrane surfaces during a reverse electrodialysis (RED) process (Figure 9) using real sea and river water streams [22]. In that study, the PCA of data acquired before, during and after long-term operation of the RED system allowed to follow the development of fouling on both anion and cation exchange membranes (AEM and CEM) in contact with both water sources and characterize the effects of fouling on membrane surfaces and of membrane cleaning efficiency, thus, allowing for membrane surface characterization and monitoring of the membrane status.

Furthermore, by using non-mechanistic modelling, it is possible to incorporate different data descriptors, including fluorescence, process operating conditions, and/or other analysis performed in real time (Figure 9). Thus, in the same RED system, PLS regression was used to model three performance parameters (pressure drop, stack electric resistance, and net power density) with and without adding as input fluorescence data acquired at membrane surfaces [23]. The study showed that fluorescence data containing information from fouling highly improved the prediction ability of the models. Additionally, by acquiring fluorescence in different membrane surfaces (anion-exchange membranes and cation-exchange membranes, river and sea sides) it was possible to confirm that fouling of anion-exchange membranes facing river water was the main factor affecting the RED stack performance.

In EMB the combination of fluorescence, current and historic process operation, was used with ANN modelling to successfully predict the state of the process [4]. That work also revealed the high relevance of operating conditions (both current and past) to the overall performance of the system, which is commonly pointed out as a weakness of mechanistic models aiming at modelling processes involving biological reactions. In a similar way, a MBR was also modelled using fluorescence data (after PCA compression), operating and analytical data as input parameters of a PLS model [24]. Five performance parameters (including COD, N and P in permeate) were successfully predicted only based on data known at real time. Furthermore, in that study the deconvolution of data with non-linear correlations was achieved through the incorporation of quadratic and interaction terms of the compressed fluorescence matrices.

Although the use of additional monitoring data may be a relevant improvement in the modelling of membrane processes, non-mechanistic modelling proved also to be suitable to predict and describe the correlations existing in different membrane processes by correlating the inputs in a more complex way than mechanistic modelling or by including additional descriptors. Operating conditions of a membrane-supported biofilm reactor for industrial effluent treatment were used alone, with elapsed time and with elapsed time plus previous operating conditions in three different modelling approaches, based on ANN, for prediction of process state [25]. Each approach proved to be suitable for different process configuration, being the process performance prediction dependent on the history of process operation for configurations resulting in the formation of an axial concentration gradient in the wastewater compartment, which was developing with time.

**Figure 10:**
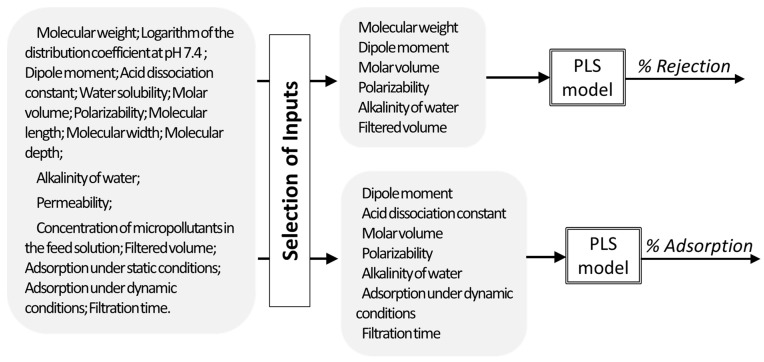
Input selection and PLS modelling of apparent rejection (% Rejection) and adsorption (% Adsorption) of micropollutants during nanofiltration of contaminated waters.

In another work [13], PLS regression was used to describe the apparent rejection and adsorption of micropollutants during nanofiltration of contaminated water streams. As inputs, physicochemical properties and molecular size parameters of the micropollutants, descriptors of the membrane and of the water matrices, and operating related conditions were used. The models developed allowed to predict the rejection and adsorption of a compound based on the properties of the compound, membrane permeability, water alkalinity, and operating conditions. Additionally, the analysis of the regression coefficients, after input selection, allowed also to understand which properties affect more the rejection and adsorption of micropollutants (being geometry of the molecule important to determine rejection, when molecules have very different geometry, while it is not so relevant for predicting adsorption) (Figure 10).

All through the examples shown, non-mechanistic modelling showed to be effective not only for estimation of performance variables in membrane processes, as well as useful in disclosing the relevance of input parameters to each output, independently of the algorithms used. Therefore, besides monitoring and characterization of membrane systems, machine learning techniques proved suitable to increase the knowledge about each system studied.

The examples described in this paper covered different membrane processes with biological reactions, micro and ultrafiltration, nanofiltration and ion-exchange membranes; however, the application of non-mechanistic models to other membrane processes is also being increasingly studied by different research groups with successful results (e.g., [26,27,28,29,30]). Kadel and co-workers studied peptide migration and selectivity during electrodialysis with filtration membranes by multivariate regression models [26,27]. Membrane distillation was modelled using ANN by Acevedo et al. [28] and by Dudchenko and Mauter [30]. The solubility of gases was predicted in nanocomposite membranes by Dashti et al. [29], using several machine learning tools. In fact, the increasing knowledge about machine learning methods (applied to several areas) and increasing number of publications, as well as the global interest and acceptability of these methods, will thus lead to increased application in several membrane processes. Furthermore, mathematical modelling, and in particular the use of machine learning methods (able to use different types of data descriptors as variables), shows to be essential and may provide further advantages when monitoring membrane process performance and fouling formation (for example to achieve online monitoring and establish anti-fouling strategies) and to optimize process performance (for example by disclosing correlations between process variables).

## 6. Strategies for Model Development

When there is no conceptual knowledge available to develop a mechanistic model, data mining and machine learning techniques are powerful tools at hand. However, even when there is not a specific need for non-mechanistic modelling techniques, the use of machine learning can be advantageous for monitoring and membrane process modelling, in particular when the mechanistic modelling is complex and there are process data available.

Machine learning can be used to extract information from complex monitoring data sets, such as spectroscopic data. This is an advantage specially when online monitoring is aimed and can be done either through the development of correlations between spectra and performance parameters (e.g., using PLS or ANN) or through unsupervised tools (e.g., PCA) to characterize the system status based on patterns (e.g., membrane surface characterization through operation and cleaning). Additionally, both non-mechanistic techniques can be combined to process data (e.g., reducing dimensionality by PCA) prior to the establishment of correlations between inputs and outputs, reducing computational effort and eliminating collinearity and noise from input data.

Furthermore, machine learning can be used to incorporate different types of process descriptors in the same tool (e.g., by using spectroscopic data and operating conditions as inputs), increasing the quality of the models achieved. In a similar way, this property can be used in hybrid modelling to improve predictions.

Ultimately, in the presence of several process descriptors, the assessment of the impact of each parameter in the model used for estimation of the output parameter allows to learn about the system and infer the mechanics involved and the relationships between inputs and outputs.

Selection of modelling tools should be based on the simplicity principle, trying first simpler structures, which may result in models with higher robustness and less overfitting. Thus, multilinear regressions (such as PLS) can be firstly assessed and, if the system presents non-linear interactions, non-linearity may be taking in account either by incorporating transformation of inputs (e.g., quadratic and interaction terms) or by using non-linear tools, such as ANN. Independently from the algorithms used for learning, the models achieved should always be properly assessed and evaluated in terms of calibration and external validation.

In a similar way, the selection of hybrid modelling structures depends on the objectives of modelling. Thus, hybrid modelling can be used not only for prediction when the mechanistic and the non-mechanistic models alone fail but also to complement information and gain knowledge about the system (e.g., to disclose missing information not integrated in mechanistic models).

## 7. Conclusions and Perspectives

Nowadays, based on the several successful studies performed for membrane processing, it is easier to look to these non-mechanistic tools with confidence, learn with the results obtained, and, above all, trust in the results for practical applications. Thus, machine learning methods may be applied to solve problems common in process implementation, such as monitoring the process performance, monitoring fouling development, and optimization of membrane processes, through meaningful predictions based on varied input data.

In fact, non-mechanistic tools proved to have the potential to translate monitoring data into process performance parameters, which can be done in real time, allowing for advanced process control. Furthermore, the ability of machine learning tools to incorporate different data sets and combine different functions (such as in hybrid modelling) is a huge advantage in the development of control tools for membrane processes. Control tools based on such modelling approaches could be used for continuous update of dynamic modelling structures, learning, and improving predictions from the online monitoring.

Finally, the approaches used to gain further knowledge from non-mechanistic models applied to membrane processes show also the potential of machine learning to promote process optimization.

## Data Availability

Not applicable.
